# Inline monitoring of cold forging processes using vibration sensors

**DOI:** 10.1038/s41598-026-49219-2

**Published:** 2026-04-16

**Authors:** Papdo Tchasse, Mathias Liewald

**Affiliations:** Institute for Metal Forming Technology, University of Stuttgart, Stuttgart, Germany

**Keywords:** Cold forging, Inline process monitoring, Vibration sensors, Unsupervised learning, Engineering, Materials science, Mathematics and computing

## Abstract

Multi-stage cold forging processes are used for the mass production of particularly resistant metal products of different level of geometrical complexity. These processes also allow a high degree of dimensional accuracy to be achieved, which can eventually exempt the formed parts from a subsequent machining treatment and therefore lower the overall production costs. Improving the robustness of cold forging processes via digitisation requires the integration of different types of sensors into the manufacturing system. However, the compactness and high geometric precision of the tool, dictated by the high loads involved during the process, and the constraints related to the quality of the final formed part, represent considerable challenges. In this context, externally attachable sensors such as vibration sensors offer a unique perspective. Therefore, this study explored the application of vibration sensors for monitoring cold forging processes. The investigated scenario involved a tool designed for the production of screw-like parts in two stages, comprising a forward rod extrusion and an upsetting stage. Different sensors with inline monitoring capabilities were attached at various locations within the manufacturing system and their recorded signals were analysed, focusing on the interdependence between the part quality, the tool load and the press activity. The results showed that vibration sensors can be used not only for a global monitoring of the press behaviour but also for the local monitoring of the tool load and the quality of the final formed part.

## Introduction

Cold forging is used in the bulk metal forming industry to manufacture small- to medium-sized parts of a mass, which usually weigh no more than 30 kg^1^. These small workpiece geometries can be manufactured in mass production with press stroke rates that can reach 300 min^− 1^^2^. In such a context, the inline and real-time process monitoring can be challenging because of the high productivity and the dynamic process environment, which is characterized by highly fluctuating thermal and mechanical loads^3,4^. Furthermore, applying sensors for the process monitoring usually involves a considerable engineering effort for tool design and it may alter the geometrical integrity of the tool. Especially the volume reduction of the tool is not desirable for cold forging applications, as the tool loads are considerably high and the pressure on the workpiece can reach up to 3000 N/mm^2^^2^, which is the reason why shrink rings are often used to reinforce the dies. In this context, a volume reduction of the tool may result in an overall stiffness reduction and hence tool weakening.

In order to mitigate the tool weakening due to the integration of sensors, recent studies addressed the application of thin, small sensors such as fibre bragg grating (FBG) and thin-film sensors. Deliktas et al. for instance integrated FBG sensors into an additive manufactured die intended for a full forward extrusion process^5^. In this study, the authors investigated the application of FBG sensors for the die temperature and strain measurement. Furthermore, thin-film sensors were used by Rekowski et al.^6^ in order to predict the concentricity of the final part in a cup backward extrusion process. In this use case, a piezoelectric thin-film disc was attached to the extrusion punch to detect concentricity deviations based on the measurement of the eccentric pressure during the forming operation. Grötzinger et al. also used a similar technology as in^6^ in order to measure the tool heating^7^. Although the use of thin-film sensors has been proven advantageous for force or temperature measurement, this technology is still expensive and its applicability in a context of industrial mass production has not been validated yet. For this reason, the forming force, for example, is still monitored via load cells^8,9^ in many applications and the tool temperature via thermocouples^10^.

A method that can be considered for an external, low-engineering monitoring of the forming process is the application of acoustic emission (AE) sensors. AE sensors have attracted significant interest for quality monitoring in various manufacturing fields such as machining^11^, casting^12^, plastic injection moulding^13^ and additive manufacturing^14^. In sheet metal forming also, Hao et al. investigated the application of AE sensors in order to monitor the dislocation motion during a tensile test^15^. A similar study was performed by Baral et al. who applied AE sensors in order to track the necking of tensile test specimens^16^. Further research was conducted by Tsuruya et al.^17^ and Yoo et al.^18^, who attempted to detect cracks during deep drawing of metal parts. Also, Ubhayaratne et al. investigated the correlation between AE signals and the wear evolution of stamping punches^19^. Although AE sensors can offer some flexibility in terms of integration and positioning within the process environment, signal filtering may be required to mitigate the interference from external, loud sources.

Taking into account the constraints of the tool robustness and the effort required for the tool design and the integration of sensors for process monitoring, this study addressed a solution that has barely been exploited in the scope of cold forging and which is based on externally attachable vibration sensors. These sensors can be attached to the press or the tool at existing threaded holes and may not require any tool modification because threaded holes are almost always given, as they are needed for the transportation of tool or press components. Some authors have previously suggested using acceleration sensors for cold forging monitoring^20–24^. However, until now, the principal focus has been on monitoring press or ram movement regardless of the quality of the final formed part. Therefore, this study provides a unique insight into the interdependence between the part quality variation, the tool load and the press activity, based on vibration measurements.

To the best of the authors knowledge, there are no or very few studies that have investigated the correlation between vibration measurements, press dynamics, tool load and part quality in the context of cold forging.

## Experimental setup

**Fig. 1:**
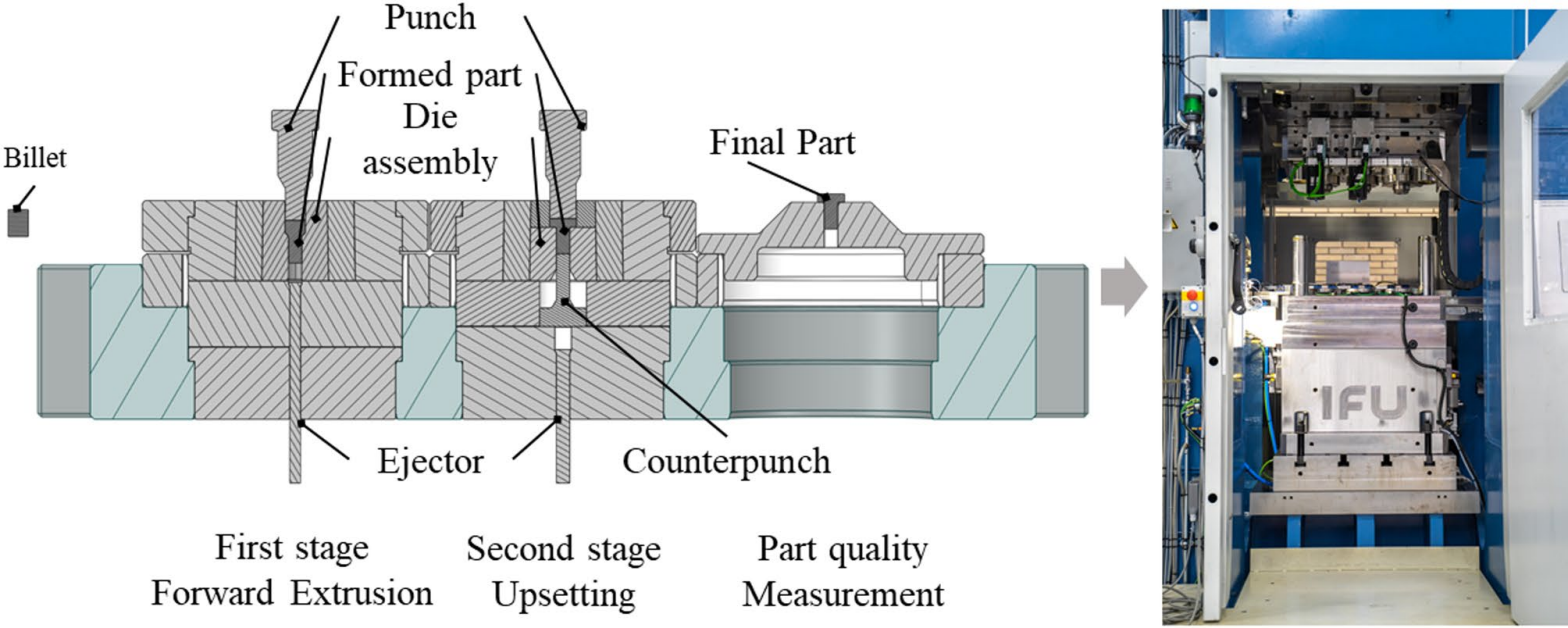
Process stages and experimental tool and press.

For this study, a two-stage cold forging process was considered, consisting of a forward rod extrusion and an upsetting stage. During the process, a billet is formed into a screw-like part without threads, drive and tip, with a head diameter of around 20 mm, head height of around 6 mm, body diameter of around 10 mm and body height of around 20 mm. The billet material used in this process was 1.5510 and the rod from which the billet was cut was drawn, annealed and trailed. The billet surface was coated with phosphate and soaped to increase temperature resistance. The tool used in this study was mounted in a servo-mechanical knuckle-joint press from Schuler with a ram upsetting capacity of 5000 kN and a stroke rate of 3–45 min^− 1^. Figure 1 shows the process stages, the tool and the press.

### Sensor concept

For this process, the head height of the final part was observed as quality criterion. In the scope of a previous study, the tool was equipped with actuators to control the vertical position of the punches, thermocouples to measure the die temperatures, a laser profiler to measure the part quality after the two process stages and force sensors to measure the forming and ejection forces^25^. The laser profiler enables an inline quality inspection of every formed part. It measures and sums up the part head height at two diametrically opposed positions. Therefore, quality information and process data can be recorded together inline without the need for further offline analysis of the produced parts.

In addition to the existing sensors, vibration sensors were prepared to be integrated into the manufacturing environment. These vibration sensors are micro-electro-mechanical systems (MEMS) that contain a suspended micro-mass, which moves perpendicularly according to the sensor’s fixation^26^. The vibration measurement is based on the capacitive principle. This means that the motion of the suspended micro-mass affects its gap distance to fixed electrodes inside the sensor body. This gap distance variation directly correlates with the capacitance of the whole assembly, which can then be measured as a change in the electric field. The micro-mass oscillation is more sensitive to motions in the sensor’s longitudinal direction and depending on the amplitude of the vibration, the sensor delivers the root mean square of the micro-mass’s moving speed in mm/s. These sensors were purchased from the company Autosen GmbH and can be used to record how quickly a measuring point deviates from a uniform or stationary motion.

**Fig. 2:**
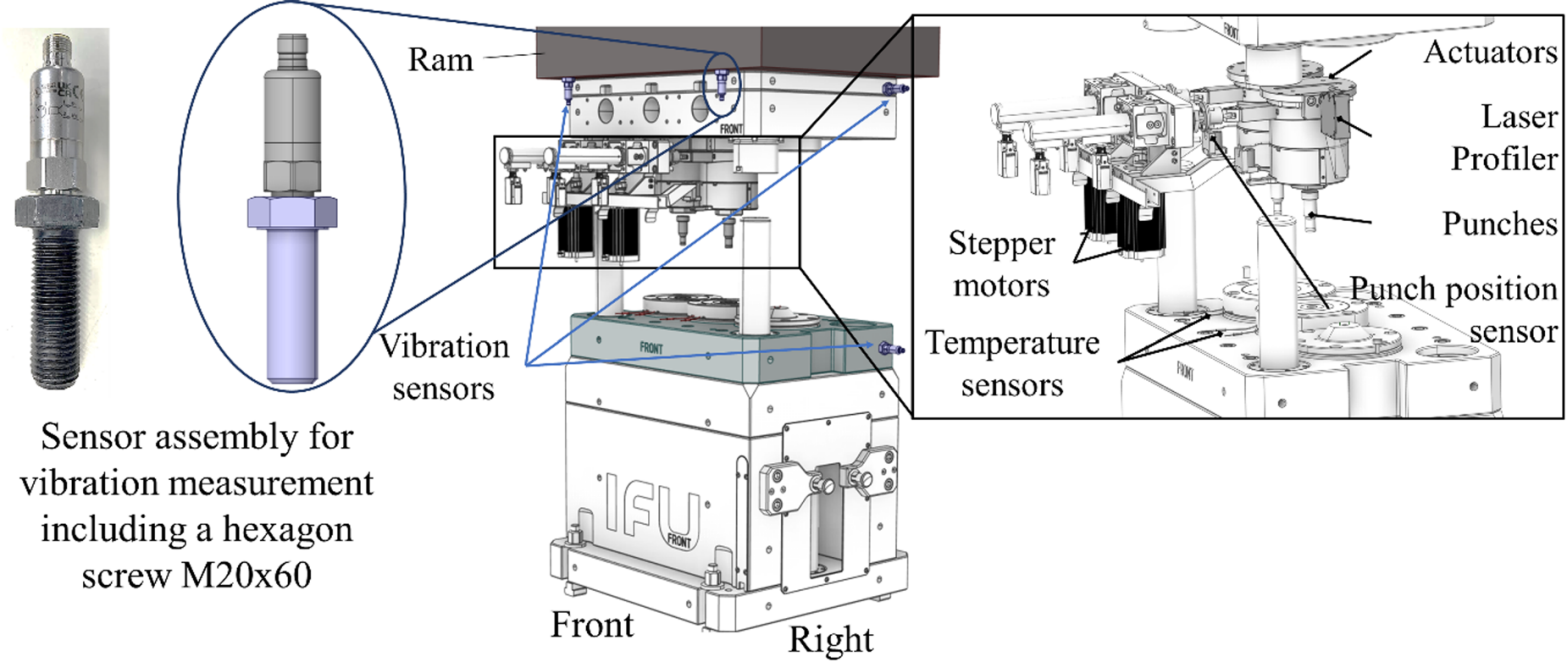
Integration concept of vibration sensors for the process monitoring.

Vibration sensors are usually applied on the external surface of the dynamical objects. For this study, in order to avoid any tool weakening, advantage was taken of existing threaded holes. These threaded holes are used for tool and ram transportation and can be found on most cold forging production components. In this use case, the threaded holes on the tool and the ram have an inside diameter of M20. For this reason, a hexagon screw with dimensions M20 × 60 was prepared as an adaptor, as the vibration sensors provide a fixation interface with M8 diameter. The resulting assembly was then attached at different positions on the ram and the tool, as shown in Fig. 2. Two vibration sensors were integrated horizontally at the back and two were integrated vertically at the front of the upper part of the tool. Finally, a further sensor was integrated horizontally on the right side at the back of the lower part of the tool. That brings the total number of vibration sensors to five.

## Results

The sensor concept was validated in this study through a continuous production run of a total of 1233 strokes. During the production, the stroke rate of the press was varied four times with given values of 25 min^− 1^, 20 min^− 1^, 15 min^− 1^ and 10 min^− 1^.

**Table 1:** Sensor position and orientation.

Sensor	Position	Orientation
Vibration Sensor 1	upper-tool-back-left	Horizontal
Vibration Sensor 2	upper-tool-front left	Vertical
Vibration Sensor 3	upper-tool-front-right	Vertical
Vibration Sensor 4	upper-tool-back-right	Horizontal
Vibration Sensor 5	lower-tool-back-right	Horizontal

Each stroke rate was maintained for approximately 300 strokes, with the punch heights varying every 25 strokes within each range. The data recording for each stroke was triggered at a ram angular position of around 120° and ended at around 280°. Vibration signals from 1 to 5 correspond to the sensor positions indicated in Table 1.

### Single press stroke

**Fig. 3:**
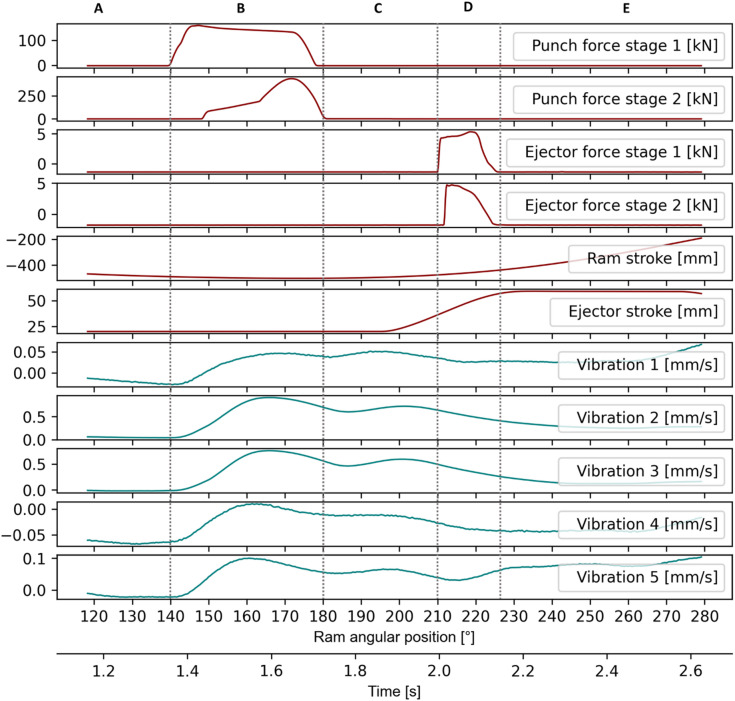
Recording of one stroke.

To analyse the vibration signals and their evolution during the forging operation, one stroke was selected that exhibits the general behaviour observed throughout the entire production run. This sample was recorded at a stroke rate of 20 min^− 1^. Figure 3 shows the different phases of the forging operation. In phase A, the ram moves towards the bottom dead center. This uninterrupted motion of the ram in one direction, which is controlled by the press servo-motor, is characterised by low oscillations of the vibration sensors.

Then, in phase B, the punches meet the part. This starts with the forward rod extrusion, followed by the upsetting stage. As soon as the workpiece is pressed and pushed into the die, the vibration amplitudes starts to rise, reaching a peak once the restraining forces caused by friction between the workpiece and the die have been overcome. Then, the oscillation frequency of vibration sensors decreases until the press ram reaches the bottom dead center. Once the bottom dead center is reached, the mechanical energy due to the press deflection and stored until then is released by a sudden acceleration of the ram upwards towards the top dead center^27^. This acceleration, which depends on the stiffness of the tool and the press, can be observed in phase C.

From phase D to phase E, the vibration signals follow the lead of the ram motion for sensors 1 to 4 that are attached to the upper tool part. Meanwhile sensor 5, which is attached to the lower tool part, seems to correlate with the ejector movements in phase D before entering a quasi-stationary state in phase E. Observing these signals, and particularly phases B, C and D, it is clear that they contain clues not only on the individual forming operations but also on the movement of the ram, which is directly connected to the servo-motor of the press. Phase B is related to the forming operation, the workpiece motion within the die as well as the corresponding frictional forces. Phase C characterizes the press and tool stiffness as well as the ram acceleration due to the press deflection. Finally, phase D describes the upward movement of the ram towards the top dead center. Generally, the vibration signals from sensors 2 and 3 display a greater amplitude range than those from the other sensors. Considering the orientation of these sensors, it appears that the vertical direction offers a greater sensitivity to the process states. However, despite the MEMS inside the sensor bodies being suspended laterally, Fig. 3 shows that the sensors can still be used to monitor lateral movements.

### Continuous production run

**Fig. 4:**
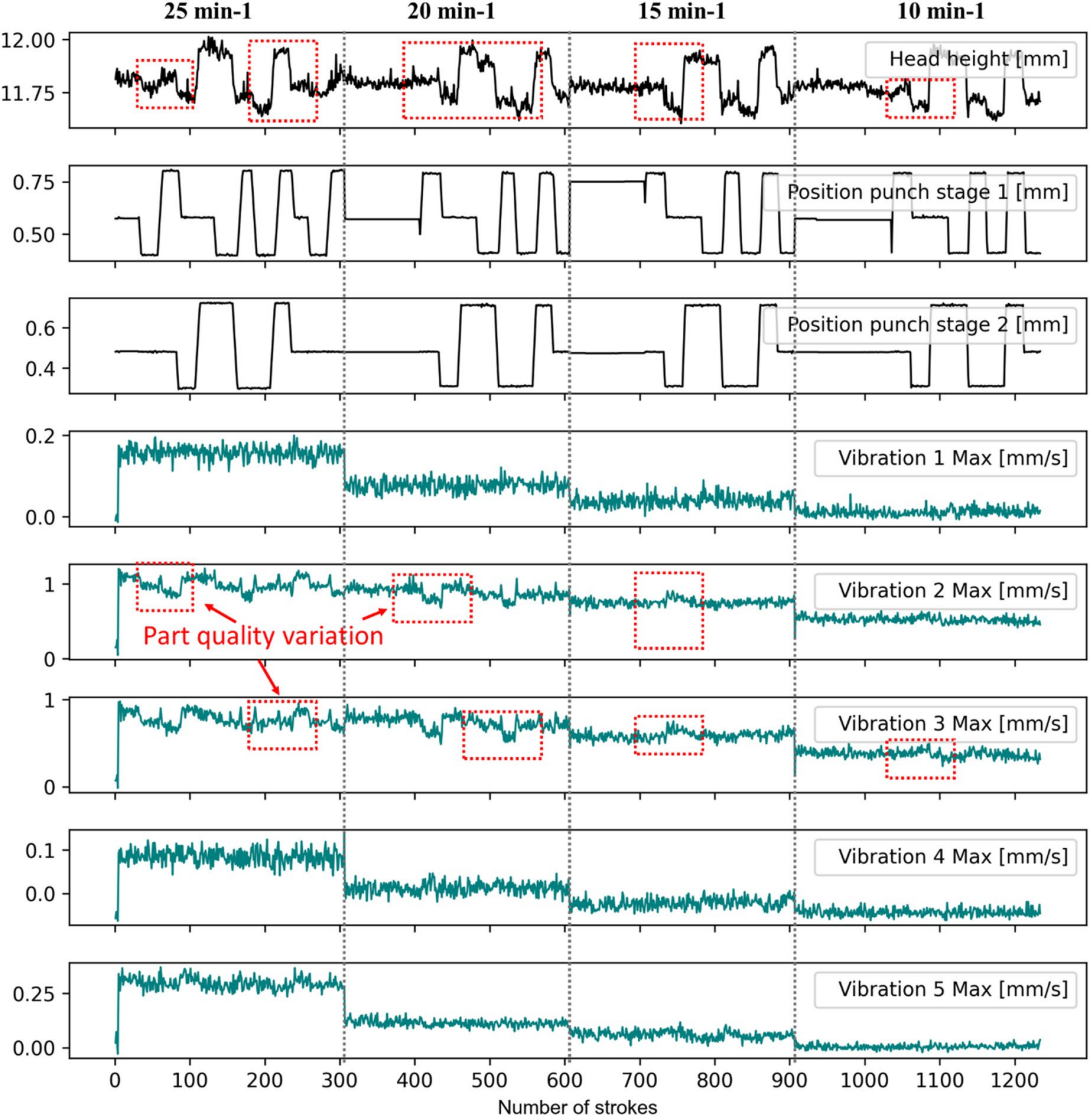
Monitoring of a continuous production of more than 1200 parts.

Figure 4 shows the measurements recorded during four sets of continuous production runs at different stroke rates and with different punch positions. For this analysis, the part quality (head height), the actuator influence (punch position) as well as the maximum values of vibration measurements for each stroke are evaluated.

At first glance, it appears that the amplitude of the vibration signals correlates directly with the stroke rates. From one stroke rate to another, the signals show a clear gradient, which for sensors 1, 4 and 5 appears sudden, whereas it is rather smooth for sensors 2 and 3. These differences in sensor signals depend not only on the sensor orientation but also on their measurement location.

The musters in Fig. 4 show that the general press behaviour can be monitored simply by observing the vibration amplitude of single press strokes. Furthermore, within each range of one single stroke rate, it appears that the vibration signals exhibit some fluctuations. These fluctuations remain within a scope that is smaller compared to the overall value range of the sensor signals. However, they seem to vary according to the process control via actuators, which directly affect the part head height, which is the part quality considered in this study. Especially the signals of sensors 2 and 3, which were placed vertically at the front of the tool, show some musters, that can be directly related to variations in part quality due to the control of the punch positions. Also, the signal amplitude of sensors 2 and 3 is generally higher than that of the other sensors. This suggests that the position and the orientation of the sensors affect the quality of the measurements. Furthermore, Fig. 4 illustrates that the sensor orientation and the corresponding sensitivity can be mapped either with the part quality variations or with the press stroke rate.

The noise in vibration signals can be explained many factors. These include mass and geometry fluctuation of the billet, billet lubricant distribution, billet fluctuating material properties, process dynamics resulting from tool tilting, tool deflection or ram upwards acceleration due to the release of the press elastic energy stored during the press motion downwards. Furthermore, these influencing factors generally affect the process differently depending on whether the process is in the ramp-up or in the steady phase, due to the resulting thermo-mechanical expansion of the production system. Some of these influencing factors may sum up or cancel each other. The investigation of these effects and their influence on the press and tool vibration would be the focus of future works.

For a further evaluation of the relationship between the part head height as part quality and the vibration measurements, the head height and each single vibration signal were correlated using a scatter plot. Each pair was then processed using k-means clustering, an unsupervised learning method. The k-means algorithm clusters the data by separating it into groups of equal variance^28^, while minimising inertia, which in this context is the within-cluster sum-of-squares criterion. For this unsupervised learning method, the number of clusters is a hyperparameter that has to be set before the model training. Although different automatic searching methods such as grid search, random search, Bayesian optimization and the Hyperband algorithm can be used for this purpose^29–31^, the number of clusters was set manually for this initial investigation based on the nature of the collected data and its observed distribution. The objective of this preliminary application of artificial intelligence was to identify the various ranges of vibration amplitude and part quality as measured by sensors and shown in Fig. 4. For this reason, the number of clusters was set to nine to capture the three prevalent part quality ranges for given stroke rates, depending on the sensor sensitivity to the changes of the stroke rate.

**Fig. 5:**
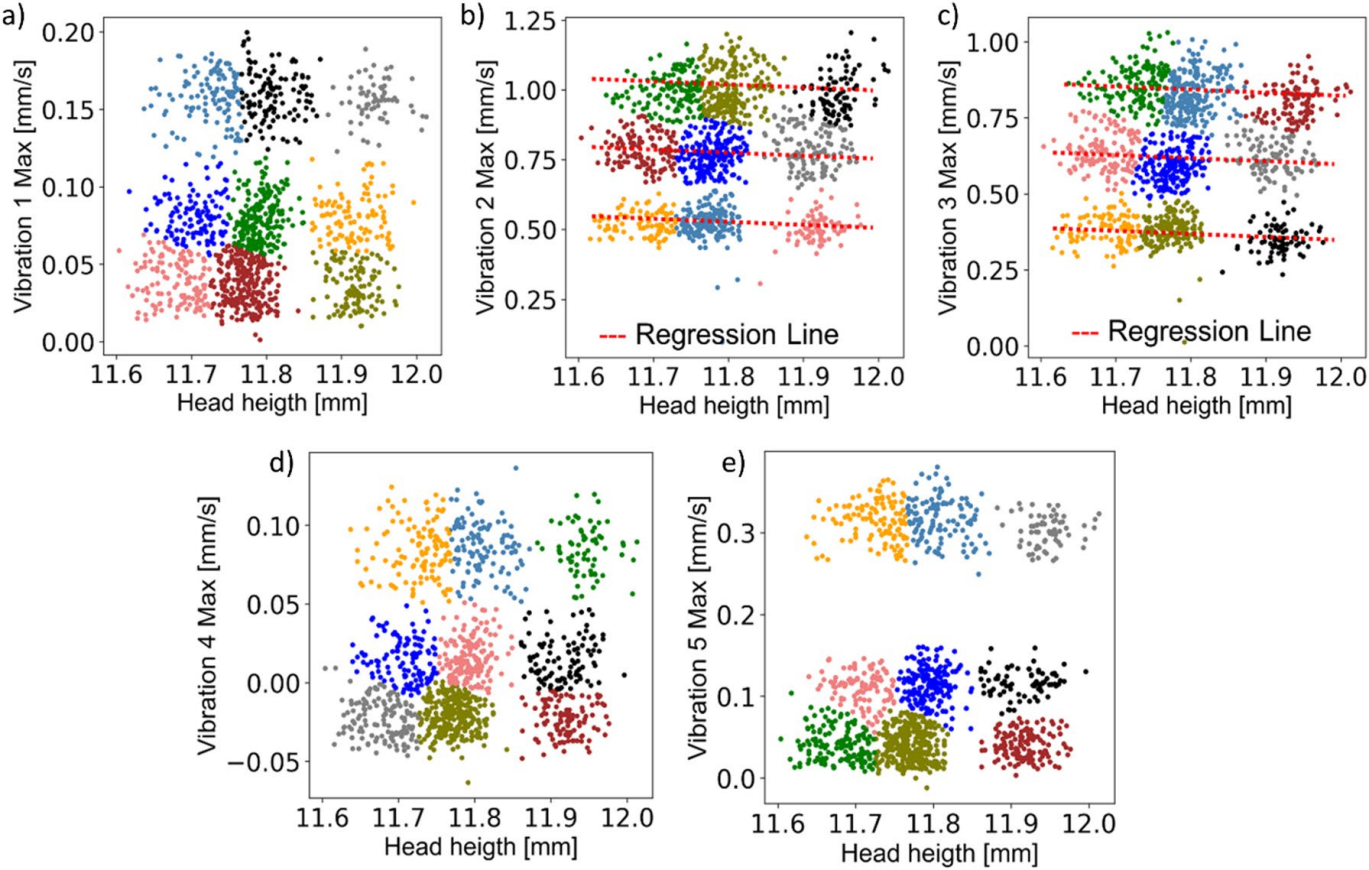
Correlation between the part quality vibration, and clusters determined using the k-means algorithm.

The correlation between the part quality, which is the part head height and the vibration measured at the tool and the ram are illustrated in Fig. 5.

Although the point clouds are scattered differently, depending on the sensors, it appears that the same classes can be identified using the k-means algorithm. The different amplitude ranges of vibration, that are related to the stroke rates can also be observed, although the transitions from 15 min^− 1^ to 10 min^− 1^ for Fig. 5a,d,e and from 25 min^− 1^ to 20 min^− 1^ for Fig. 5b and c are not clearly highlighted. The variations induced by the changes in part quality are noticeable at a glance and were also highlighted via the k-means algorithm. Of course, the data spread in Fig. 5a,d,e suggests a relatively high variance compared to the Fig. 5b,c. However, for all the cases, it clearly appears that the change of the part quality can easily be monitored using an envelope curve by setting permissible value limits and tolerances. A linear correlation between vibration signals and part quality values can even be identified in the case of Fig. 5b,c, which confirms that the minimal changes in the part quality can be captured by using externally attachable vibration sensors.

The increase or decrease of the punch positions while keeping the same ram stroke provokes respectively a decrease or an increase of the forming force. This results in a change in the forming work, which directly affects the amount of heat energy released by mechanical dissipation within the crystal lattice, and the heat energy released by friction between the workpiece and the die^32^. The corresponding heat was measured using thermocouples integrated inside the dies.

**Fig. 6:**
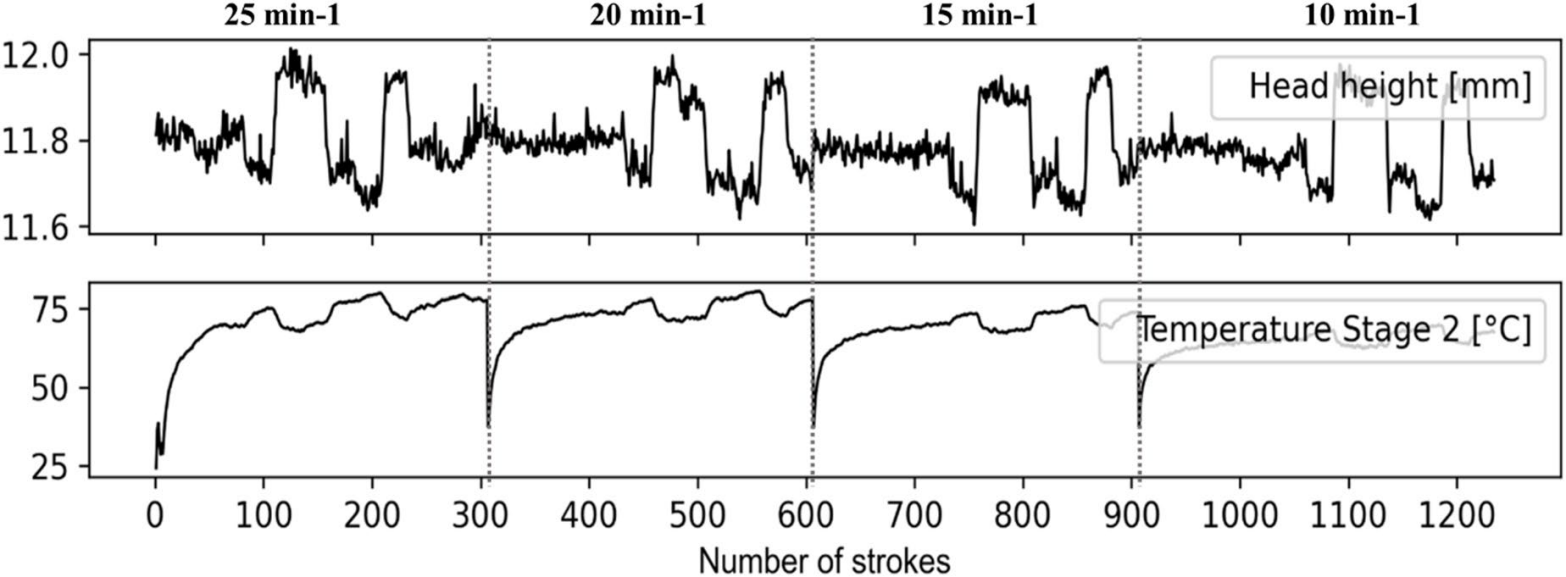
Impact of the part quality variation on the die temperature.

The measurement of one of the thermocouples is shown in Fig. 6 and illustrates how a variation in part quality that affects the vibration signals also impacts the tool temperature. These results demonstrate that an increase in the part head height correlates with a decrease in die temperature and reciprocally, if the temperature decreases, the head height of the final formed part increases.

## Conclusion

The final part of a cold forging process is the result of the interactions between various influencing factors that are related to the workpiece itself, the tool and the press. For monitoring purposes, observing these influencing factors often involves a considerable effort for tool design and sometimes a tool weakening, which, considering the high loads inherent to cold forging, is undesirable. To address this issue, vibration sensors provide a valuable perspective, which until now has barely been investigated in the scope cold forging. These sensors can be attached externally to the press or the tool, at existing threaded holes that are usually intended for transportation purposes. Analysing the measurements recorded with such sensors confirmed the interdependence and the correlation between the part quality, the tool load and the press activity. The results showed that not only the quality of the final forged part but also the press activity can be monitored without the known expenditure related to sensor integration in cold forging. The results of the investigations showed that a simple amplitude monitoring by setting an envelope curve can help to detect stroke rate disturbances and part quality fluctuations. Further works would involve exploiting such vibration measurements in order to compensate detected process disturbances by using closed-loop control strategies. Furthermore, an application of artificial intelligence or machine learning methods combined with closed loop control techniques would enhance process transparency and enable long-term, robust control of the cold forging process.

## Data Availability

Due to the sensitive nature of the research supporting data and the ongoing works, the datasets used and/or analysed during the current study will be available from the corresponding author only upon reasonable request.
